# Maternal 27-hydroxycholesterol concentrations during the course of pregnancy and in pregnancy pathologies

**DOI:** 10.1186/s12884-017-1287-7

**Published:** 2017-04-04

**Authors:** Brigitte Sophia Winkler, Ulrich Pecks, Laila Najjari, Nicola Kleine-Eggebrecht, Nicolai Maass, Markus Mohaupt, Geneviève Escher

**Affiliations:** 1 Department of Obstetrics and Gynecology, University Hospital of RWTH Aachen, Pauwelsstraße 30, 52074 Aachen, Germany; 2 Department of Nephrology, Hypertension and Clinical Pharmacology, Inselspital, Department of Clinical Research, University of Bern, Freiburgstrasse, 3010 Berne, Switzerland; 3 Department of Obstetrics and Gynecology, University Hospital of Schleswig-Holstein, Michaelisstraße 16, 24105 Kiel, Germany

**Keywords:** 27-hydroxycholesterol, 27-OHC/TC ratio, Preeclampsia, HELLP-syndrome, Intrauterine growth restriction, Intrahepatic cholestasis in pregnancy, Lipid metabolism

## Abstract

**Background:**

The oxysterol 27-hydroxycholesterol (27-OHC) plays an important role in the regulation of cholesterol homeostasis. Pregnancy pathologies like preeclampsia (PE), HELLP-syndrome (HELLP), intrauterine growth restriction (IUGR) and intrahepatic cholestasis in pregnancy (ICP) are linked to disturbances in lipid metabolism. In the present study, we hypothesized a specific gestational regulation of 27-OHC and compromised 27-OHC levels due to placental and hepatic diseases in pregnancy resulting in a dysregulation of lipid metabolism.

**Methods:**

The 27-OHC was measured by gas-chromatography-mass spectrometry (GC-MS) and related to cholesterol concentrations. In the longitudinal cohort, a complete set of samples of healthy patients (n = 33) obtained at three different time points throughout gestation and once post-partum was analyzed. In the cross sectional cohort, patients with pregnancy pathologies (IUGR *n* = 14, PE *n* = 14, HELLP *n* = 7, ICP *n* = 7) were matched to a control group (CTRL) of equal gestational ages.

**Results:**

The 27-OHC levels already increased in the first trimester despite lower TC concentrations (*p* < 0.05). During the course of pregnancy, a subtle rise in 27-OHC concentrations results in an overall decrease of 27-OHC/TC ratio in between the first (*p* < 0.05) and second trimester. The ratio remains stable thereafter including the post-partum period. No significant differences have been observed in pregnancy pathologies as compared to the CTRL group.

**Conclusion:**

In conclusion, 27-OHC may have a compensatory role in cholesterol metabolism early in pregnancy. The conserved 27-OHC/TC ratio in pregnancy pathologies suggest that neither the placenta nor the liver is majorly involved in the regulation of 27-OHC metabolism.

## Background

Cholesterol is an essential structural component of cell membranes and is required to establish proper membrane permeability and fluidity. In addition to its importance within cells, cholesterol also serves as a precursor for the biosynthesis of bile acids, vitamin D, and steroid hormones. Thus, a tight regulation of cholesterol availability is crucial at each developmental stage in both, the fetus and the mother. Human pregnancy is a state of hyperlipidemia [1]. Maternal cholesterol concentrations increase by 25 to 50%, thus contributing to the anabolic state of the mother during pregnancy. Some pregnancy pathologies are linked to disturbances in lipid metabolism and cholesterol homeostasis. Women with intrahepatic cholestasis of pregnancy (ICP) show increased cholesterol and LDL levels [2], while cholesterol and LDL concentrations of women suffering from intrauterine growth restriction (IUGR) are lower as compared to gestational age matched controls (CTRL) [3]. Moreover, preeclampsia (PE) has been regarded as a state of dyslipidemia with a prevalence of small dense LDL particles and high triglyceride concentrations [4, 5].

Oxysterols are mono-oxygenated metabolites of cholesterol and play an important role in the regulation and maintenance of whole-body cholesterol homeostasis. They participate in different aspects of lipid metabolism and have been ascribed a number of roles in connection with atherosclerosis, inflammation, apoptosis and immunosuppression [6, 7].

27-hydroxycholesterol (27-OHC) is the quantitatively most dominating oxysterol in the circulation of human adults [8]. It is produced upon hydroxylation of cholesterol by the enzyme sterol 27-hydroxylase (CYP27A1) [9]. In the liver, CYP27A1 catalyzes the first step of the alternative bile acid pathway and intermediate step in the classic bile acid pathway [10, 11]. In extra-hepatic tissues, CYP27A1 has been implicated in cholesterol efflux [12–14], the first and rate limiting step of reverse cholesterol transport. Thus, the particularly high expression of CYP27A1 in vascular endothelial cells [15] and macrophages [9] can be considered as a key element for the removal of cholesterol excess. Loss of function mutation in CYP27A1 leads to Cerebrotendinous Xanthomatosis, a disease characterized by the absence of 27-OHC and associated to early onset of atherosclerosis, despite normal circulating plasma cholesterol concentrations.

In vitro, 27-OHC is a potent inhibitor of cholesterol synthesis, as it acts as negative feedback regulator of the HMGCoA-reductase, the rate limiting enzyme of cholesterol biosynthesis [16]. Furthermore, 27-OHC is a physiologically relevant endogenous agonist of the liver-X-receptor (LXR), thereby governing the transcription of genes involved in cholesterol catabolism, transport and elimination [17, 18]. During pregnancy, oxysterols are suggested to impair differentiation and invasion of trophoblast cells via the LXR [19, 20].

Though 27-OHC has various physiological functions that might influence pregnancy outcome, limited attention has been paid to concentration changes in pregnant women so far. In the present study, we hypothesized a specific gestational regulation of 27-OHC and compromised 27-OHC levels due to different placental and hepatic diseases in pregnancy resulting in a dysregulation of lipid metabolism. We first aimed to measure the cholesterol composition in serum throughout pregnancy and post-partum (p.p.), and second to compare women without and those suffering from gestational diseases (ICP, PE, HELLP, IUGR). As suggested by Martin et al 1997, we related the 27-OHC concentrations to total cholesterol concentrations (TC) to adjust for inter-individual variability and to get a proxy of CYP27A1 activity.

## Methods

Ethical approval was obtained from the ethic committee of the medical faculty of the Technical University of the Rheinisch Westfälisch Technische Hochschule (RWTH) Aachen (EK 138/06 19.06.2006, EK 119/08 02.09.2008, EK 154/11 19.07.2011). Written and informed consent was obtained from each patient. Patients were recruited and serum was sampled specifically to analyze biomarkers of pregnancy and pregnancy pathologies. Samples were aliquoted and stored at the Aachen biobank at -80 °C till further use (2006 to 2012). Sera to be analyzed were then chosen from the biobank according to the following criteria.

In accordance with the ISSHP criteria [21] PE was defined as new onset of hypertension >140/90 mmHg after 20 weeks of gestation and proteinuria >300 mg/d or at least 2+ in urine dip-stick.

IUGR was defined in accordance with the ACOG guidelines [22]. The following criteria had to be fulfilled: estimated fetal weight <10th percentile in addition to (i) deceleration of fetal growth rate >40 percentiles in serial measurements, (ii) elevated resistance index in umbilical artery Doppler sonography above the 95th percentile, or absent or reversed end diastolic blood flow, (iii) fetal asymmetry (head-to-abdominal circumference ratio >95th percentile), or (iv) oligohydramnios (amniotic fluid index <6 cm).

HELLP syndrome was defined as published recently [23] as the presence of upper abdominal pain in accordance with the confirmation of the following laboratory criteria in maternal serum analyses: hemolysis (haptoglobin levels <30 mg/dL), elevated liver enzyme activity (AST, ALT, LDH) three standard deviations above the mean, and platelet count <100.000 G/L.

ICP was defined as recently described by Mays et al. 2010 as the clinical finding of pruritus in combination with an increased bile acid concentration in maternal serum analysis >14 μmol/L, and elevated liver enzyme activity (AST, ALT) above the reference values (>35 U/L) [24] when HELLP syndrome was excluded.

Exclusion criteria for case and control groups were multiple gestation, abnormal fetal karyotype, fetal anomalies, patients with clinical or biochemical signs of infection, positive TORCH screening results, maternal diabetes mellitus or other severe maternal metabolic disorders, preterm birth following complications other than IUGR, PE, or HELLP syndrome, and a patient’s withdrawal from the study. Samples were taken from women prior to or at least 5 days after administration of corticosteroids (betamethasone) for induction of fetal lung maturity when clinically indicated to avoid interference by steroid hormone application to the mother.

Longitudinal cohort: Serum of 33 healthy patients, who delivered after 37 weeks of gestation, was taken at three different time points throughout gestation and at one time point p.p. and analyzed by GC-MS (in total 132 samples). The age range of participants in the longitudinal group was from 21.5 to 48 years with a mean maternal age of 30.7 years.

Cross sectional cohort: In the Aachen pregnancy serum biobank, 7 patients were identified as ICP with an age range from 22.5 to 38.1 years and a mean maternal age of 25.8 years. 7 patients were classified as HELLP with an age range from 27.4 to 43 years and a mean maternal age of 32.4 years. 14 patients were IUGR without hypertension (7 delivered before 34 weeks, 7 after 34 weeks) with an age range from 22.0 to 40.3 years and a mean maternal age of 30.4 years. 14 patients were PE without signs of IUGR (7 delivered before 34 weeks, 7 after 34 weeks) with an age range from 21.8 to 41.5 years and a mean maternal age of 31.1 years. All of these patients with complicated pregnancies were matched to a control group (CTRL) of equal gestational ages consisting of 42 women.

### Lipid-analysis

The 27-OHC was measured in plasma (100-1000 μl) by gas-chromatography-mass spectrometry (GC-MS) as described previously [25] with 100 ng 5α-cholestan-3β,6α-diol and 100 ng stigmasterol as standards. A standard curve with increasing known concentrations was performed for each extraction panel. Inter-assay variability was controlled using an aliquot of plasma pool.

Serum lipid profile including triglycerides (TG), TC, LDL-C and HDL-C was performed by colorimetric enzymatic methods using an automated photometric measuring unit (Roche/Hitachi Modular P800, Roche Diagnostics, Basel, Switzerland; triglycerides GPO-PAP reagent, cholesterol CHOD-PAP reagent, LDL-C plus 2nd generation reagent, HDL-C plus 3rd generation reagent, Cobas®, Roche/Hitachi, Mannheim, Germany). Measurement ranges were: TG = 4–1000 mg/dL, TC = 3–800 mg/dL, LDL-C = 3–550 mg/dL, and HDL-C = 3– 120 mg/dL.

Data analysis was carried out using the statistical program Prism Version 5 Software (GraphPad Software Inc., CA, USA). Clinical data are presented as means and 95% confidence interval (95% CI) if metric. Correlations were analyzed by Spearman’s correlation coefficient. Correlation coefficient values were considered significant if *p*-value was <0.05. Kruskal-Wallis test followed by Dunn’s comparison post hoc test was conducted for comparison of measured variables between the groups where indicated. Values of p < 0.05 were regarded as significant.

## Results

**Fig. 1:**
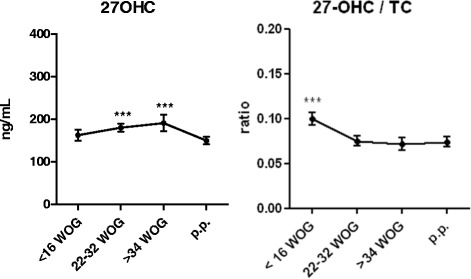
27-OHC levels and the 27-OHC/TC-ratio throughout gestation and post-partum in the longitudinal cohort. As compared to the first trimester mean 27-OHC levels increased during pregnancy by 11% in the second trimester and 18% in the third trimester and dropped down by 8% post-partum. 27-OHC: 27-hydroxycholesterol. TC: total cholesterol. WOG: week of gestation. p.p.: post-partum. Correlations were analyzed by Spearman’s correlation coefficient. Correlation coefficient values were considered significant if p-value was <0.05. Kruskal-Wallis test followed by Dunn’s comparison post hoc test was conducted for comparison of measured variables between the groups where indicated. Values of *p* < 0.05 were regarded as significant

In the longitudinal cohort of 33 patients with uncomplicated pregnancies blood samples were obtained at 10+/-5, 27+/-5, and 37+/-3 weeks of gestation. A sample taken at 13+/-9 weeks p.p. represented non-pregnant control conditions. Clinical characteristics and basic lipid profiles are presented in Tables 1 and 2. Both 27-OHC levels and the ratio of 27-OHC to TC throughout gestation and p.p. are presented in Fig. 1. As compared to the first trimester mean 27-OHC levels increased during pregnancy by 11% in the second trimester and 18% in the third trimester and dropped down by 8% p.p. (Fig. 1). Mean TC levels increased by 63% from first to third trimester, followed by a drop of 28% p.p.level (Table 2). TC p.p. was still higher than during the first trimester (Table 2). The 27-OHC/TC ratio, indicating apparent CYP27A1 activity, was 26% higher in first trimester as compared to second and third trimester and the p.p. state (Fig. 1).

**Table 1:** Clinical characteristics of the women with uncomplicated pregnancy in the longitudinal cohort

	Mean or %	Lower 95% CI	Upper 95% CI
Maternal age (y)	30.7	28.7	32.7
Maternal BMI (kg/m^2^)	24.0	22.1	26.0
Primiparity (%)	85		
Actual smoking status (%)	15		
Mode of delivery (% c-section)	25		
Fetal gender (% female)	55		
WOG at delivery (w)	40.2	39.8	40.6
Neonatal birth weight (g)	3536	3380	3692
Neonatal weight percentile	50.5	41.7	59.2
Gestational age at first blood collection (w)	9.9	8.9	10.9
Gestational age at second blood collection (w)	26.1	25.3	27.0
Gestational age at third blood collection (w)	36.5	36.0	37.0
Weeks post partum at blood collection (w)	12.5	20.6	4.5

*WOG* week of gestation

**Table 2:** Basic lipid profiles of the women with uncomplicated pregnancy in the longitudinal cohort

	< 16 WOG			22 - 32 WOG			> 34 WOG			p.p.		
Lipids	Mean or %	Lower 95% Cl	Upper 95% Cl	Mean or %	Lower 95% CI	Upper 95% CI	Mean or %	Lower 95% CI	Upper 95% CI	Mean or %	Lower 95% CI	Upper 95% Cl
TC	165.3	154.2	174.2	245.7	230.5	260.9	268.8	254.8	282.8	204.9	189.8	220.0
LDL	83.39	74.78	92.01	139.9	126.2	153.6	154.5	140.6	168.3	116.7	105.2	128.2
HDL	59.15	54.61	63.70	77.85	71.90	83.79	71.30	65.47	77.14	62.85	57.51	68.19
TG	90.94	76.17	105.7	172.0	147.7	197.3	255.2	217.4	293.1	107.3	87.74	126.9

*WOG* week of gestation, *TC* total cholesterol, *LDL* low density lipoprotein, *HDL* high density lipoprotein, *TG* triglycerides

The clinical characteristics and basic lipid profile of the cross-sectional study groups are presented in Table 3. Mean maternal age was similar in CTRL, PE, IUGR, and ICP, while mothers with HELLP tended to be older. About 20% of mothers in the IUGR group and in the CTRL group smoked during pregnancy while none of the patients in the other groups smoked. Mothers with pregnancy pathologies gave birth earlier compared to the CTRL group as indicated by the non-overlap of the 95% CIs. In the ICP group mothers were more likely to deliver girls. Mean gestational ages of the study groups at blood sampling were kept similar to the CTRL counterparts.

**Table 3:** Clinical characteristics and basic lipid profiles of the women with gestational diseases in the cross-sectional study group

	CTRL			PE			IUGR			ICP			HELLP			*P*-value
	Mean or %	Lower 95% CI	Upper 95% CI	Mean or %	Lower 95% CI	Upper 95% CI	Mean or %	Lower 95% CI	Upper 95% CI	Mean or %	Lower 95% CI	Upper 95% CI	Mean or %	Lower 95% CI	Upper 95% CI	Group
Maternal age (y)	30.1	28.2	31.9	31.1	27.9	34.3	30.4	27.1	33.7	31.7	25.8	37.7	37.0	32.4	41.6	0,2272
Maternal BMI (kg/m^2^)	24.9	22.8	26.9	26.0	23.5	28.6	22.3	19.8	24.8	23.6	19.8	27.3	25.6	17.7	33.6	0,3151
Primiparity (%)	50			57			64			71			57			0,875
Actual smoking status (%)	19			0			21			0			0			0,2501
Gestational age at blood collection (w)	33.5	31.9	35.1	33.7	30.8	36.5	32.2	28.6	35.7	35.1	31.6	38.5	33.5	28.7	38.2	0,9601
Systolic blood pressure (mmHg)	117.5	113.8	121.3	149.3	144.2	154.4	119.6	107.7	131.5	120.1	109.4	130.9	154.1	133.2	175.1	<0.0001
Diastolic blood pressure (mmHg)	67.2	64.4	70.0	93.8	90.4	97.2	68.6	60.7	76.4	71.0	64.1	77.9	91.3	83.7	98.8	<0,0001
WOG at delivery (w)	39.4	39.0	39.7	34.5	31.7	37.2	32.5	29.0	36.1	37.7	36.6	38.8	33.5	28.7	38.2	<0,0001
Mode of delivery (% c-section)	40			86			86			43			100			0,0013
Fetal gender (% female)	57			43			57			100			71			0,2083
Neonatal birth weight (g)	3369	3255	3483	2181	1622	2741	1348	813	1882	3123	2715	3530	1985	1147	2823	<0,0001
Neonatal weight percentile	47.3	39.6	55.0	27.9	16.8	39.1	2.1	1.4	2.9	54.2	28.0	80.4	24.6	5.3	43.9	<0,0001
TC	267.7	252.0	283.4	260.7	224.2	297.2	223.9	202.8	244.9	317.7	241.3	394.1	222.9	189.5	256.2	0,0131
LDL	150.5	137.6	163.4	144.9	117.3	172.5	111.6	94.01	129.3	206.0	141.7	270.3	111.6	84.04	139.1	0,0015
HDL	76.88	70.76	83.00	65.14	51.84	78.44	78.00	66.95	89.05	56.86	52.71	61.01	70.29	43.23	97.34	0.0356
TG	222.2	197.6	246.8	317.1	245.6	388.5	174.2	137.4	211.0	261.9	212.5	311.2	293.3	124.5	462.1	0,0121

Mean maternal age was similar in CTRL, PE, IUGR, and ICP, while mothers with HELLP tended to be older. About 20% of mothers in the IUGR group and in the CTRL group smoked during pregnancy while none of the patients in the other groups smoked. Mothers with pregnancy pathologies gave birth earlier compared to the CTRL group as indicated by the non-overlap of the 95% CIs. In the ICP group mothers were more likely to deliver girls. Mean gestational ages of the study groups at blood sampling were kept similar to the CTRL counterparts. As estimated by non-overlap of 95% CI TC and LDL levels were lower in IUGR by 17% and 25%, respectively, as compared to the CTRL group. A similar trend can be observed in the HELLP syndrome. In ICP HDL levels are 25% lower while LDL tended to be 37% higher as compared to CTRL. We found a tendency of higher TG values (+43%) in the PE group in comparison to CTRL. CTRL: control group. PE: preeclampsia. IUGR: intrauterine growth restriction. ICP: intrahepatic cholestasis in pregnancy. HELLP: hemolysis, elevated liver enzymes, low platelets syndrome

The basic lipid profiles differed between the study groups. As estimated by non-overlap of 95% CI TC and LDL levels were lower in IUGR by 17% and 25%, respectively, as compared to the CTRL group. A similar trend can be observed in the HELLP syndrome. In ICP HDL levels are 25% lower while LDL tended to be 37% higher as compared to CTRL. We found a tendency of higher TG values (+43%) in the PE group in comparison to CTRL.

**Fig. 2:**
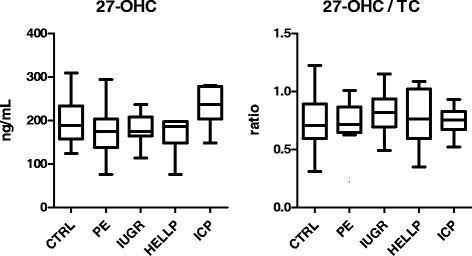
27-OHC levels and the 27-OHC/TC-ratio of women with gestational diseases in the cross-sectional study group. No significant differences were observed between any of the study groups and the CTRL. CTRL: control group. PE: preeclampsia. IUGR: intrauterine growth restriction. ICP: intrahepatic cholestasis in pregnancy. HELLP: hemolysis, elevated liver enzymes, low platelets syndrome. 27-OHC: 27-hydroxycholesterol. TC: total cholesterol. Correlations were analyzed by Spearman’s correlation coefficient. Correlation coefficient values were considered significant if *p*-value was <0.05. Kruskal-Wallis test followed by Dunn’s comparison post hoc test was conducted for comparison of measured variables between the groups where indicated. Values of *p* < 0.05 were regarded as significant

TC-, 27-OHC- levels and 27-OHC/TC-levels ratios are presented in Fig. 2. No significant differences were observed between any of the study groups and the CTRL. Nevertheless, 27-OHC tended to be higher in ICP (Fig. 2).

## Discussion

In this study, we quantified for the first time the oxysterol 27-OHC at different stages of gestation, analyzed the impact of different diseases with placental dysfunction or maternal hepatic conditions and calculated the 27-OHC/TC ratio, a marker for CYP27A1 activity.

During pregnancy, lipid concentrations increase steadily and pathologic pregnancies are associated with altered lipid profiles. In this study, we quantified the oxysterol 27-OHC, a hydroxylation product of cholesterol by the enzyme CYP27A1. The amount of 27-OHC increases throughout gestation and decreases p.p. to the levels found in the first term of pregnancy. Interestingly, the changes in 27-OHC are not exactly parallel to those found in TC, as indicated by the 27-OHC/TC ratio. 27-OHC concentrations are relatively higher than TC in first trimester samples, while in later phases of gestation and also p.p. both cholesterol levels were parallel to each other. Based on our results, a relevant involvement of impaired CYP27A1 expression in pregnancy diseases associated with placental and hepatic dysfunction can be ruled out. The amount of serum 27-OHC was not changed in PE, IUGR and HELLP and was slightly increased in ICP. Since the 27-OHC/TC ratio remains unchanged, a role for impaired CYP27A1 activity can be excluded.

The expected potent biological activities of the oxysterol 27-OHC in cholesterol homeostasis are first to suppress cholesterol biosynthesis by negative feedback regulation of the rate-limiting enzyme of cholesterol biosynthesis, 3-hydroxy-3-methylglutaryl-coenzyme A reductase, and second to provide an intermediate step in bile acids biosynthesis, a major route for cholesterol elimination. The production and the transport of 27-OHC from extrahepatic tissues to the liver might also be an alternative pathway for reverse cholesterol transport based on HDL. Weingartner suggested, that the production and the transport of 27-OHC from extrahepatic tissues to the liver may be an alternative pathway for reverse cholesterol transport based on HDL [26]. Our results suggest an adaptation of CYP27A1 activity to increase the production of 27-OHC in order to overcome the increased cholesterol concentrations, even in ICP conditions. This observation highlights that the liver is still able to synthesize 27-OHC. Additionally, these results are in line with other reports suggesting that 27-OHC production is preserved in patients with liver diseases [27].

Of interest, 27-OHC levels already increase in the first trimester despite lower TC concentration as a result of a high consumption and the luteo-placental shift during early pregnancy [28]. This might either be a compensatory mechanism to lower cholesterol levels [8, 9], or a preparation for the upcoming high cholesterol levels in later phases of pregnancy. It may have additional regulatory roles as oxysterols have been shown to affect trophoblast invasion via LXR-dependent pathways [29] and hence potentially influence pregnancy outcome.

With ongoing pregnancy, TC increases. This can be attributed to increased cholesterol synthesis or a lower cholesterol catabolism [30]. In our study, the increased TC is accompanied by a subtle rise in 27-OHC concentrations, resulting in an overall decrease of the 27-OHC/TC ratio from first to second trimester. This suggests that 27-OHC is not necessarily involved in the inhibition of cholesterol catabolism once pregnancy has reached the second trimester. The ratio remains stable after the first trimester until term and up to the post-partum period, suggesting a highly balanced cholesterol-oxysterol homeostasis throughout pregnancy.

Little is known about the concentrations of 27-OHC during pregnancy pathologies. Moon et al. observed a 1.4 fold increase in 27-OHC concentrations in PE compared to a control group while TC levels were 1.4 lower. Our results do not support a role for 27-OHC in the pathomechanism of PE. Several reasons can be proposed to explain these differences. First, Moon et al. chose a control group consisting of patients that gave birth preterm for other reasons than PE [31]. As preterm birth per se has been linked to hyperlipidemia [32], this – in our view – is not a reliable control group. Second, there is often a mix with patients suffering from IUGR in addition to PE. In our study, we have clearly separated IUGR from the PE group. IUGR is associated with a decreased concentration of TC [33, 34]. Thus, it is conceivable that in a subgroup of PE with IUGR TC may decrease with increasing levels of 27-OHC. In the present study, the 27-OHC/TC ratio was not different between the IUGR and the CTRL group, respectively, at the onset of the disease. Finally, the conflicting data could simply be attributed to the higher body mass index (in Moon’s study) or to the different genetic background of the study population.

In our study, pregnancies associated with placental insufficiency like PE and IUGR were not associated with alterations of 27-OHC levels, which may be explained by the low expression of CYP27A1 in placenta. In the ICP group with increased TC, 27-OHC was slightly but not significantly increased. This rather indicates that CYP27A1 is not impaired in this condition. As serum bile acids are increased, we can only assume that the classic pathway of bile acids initiated by CYP27A1 is enhanced as protective mechanism to decrease TC. Thus, the increased circulating TC in ICP can be explained by enhanced hepatic cholesterol synthesis or increased reverse cholesterol transport.

Strengths of this study are the evaluation of the time course of 27-OHC and CYP27A1 activity during physiological pregnancies and in non-pregnant conditions at about 13 weeks p.p., combined with a cross-sectional cohort for the investigation of pregnancy pathologies with clearly defined subgroups and with an appropriate matched control group. Additionally, we measured 27-OHC levels at disease onset when most pronounced changes to normal pregnancy would have been expected. It remains speculation whether 27-OHC may play a role in the initiation of pregnancy pathologies earlier in gestation. These results have to be verified in a larger cohort of patients.

## Conclusions

In conclusion, 27-OHC may play a compensatory role in cholesterol metabolism early in pregnancy while cholesterol-oxysterol-homeostasis is highly controlled during the second and third trimester, and resembles the non-pregnant situation. The conserved 27-OHC/TC ratio in pregnancy pathologies suggests that neither an impaired placental function nor the change in hepatic function, as those found in ICP, account for the changes in circulating 27-OHC concentrations. It is conceivable that 27-OHC plays a role in the regulation of the cholesterol metabolism during early pregnancy. In late stages of pregnancy diseases, 27-OHC appears not to be altered. Yet, it is still conceivable that in the first trimester, where oxysterols may impact trophoblast invasion, placental vasorelaxation and inflammation, a deranged 27-OHC could be linked to the initiation of pregnancy pathologies. Hence, further investigations are required with the focus on early pregnancy before the manifestation of the disease.

## Abbreviations

27-OHC: 27-hydroxycholesterol
CTRL: Control group
CYP27A1: Sterol 27-hydroxylase
HELLP: Hemolysis, elevated liver enzymes, low platelets syndrome
ICP: Intrahepatic cholestasis in pregnancy
IUGR: Intrauterine growth restriction
p.p.: Post-partum
TC: Total cholesterol concentrations
